# Transcriptome analysis of skin fibroblasts with dominant negative *COL3A1* mutations provides molecular insights into the etiopathology of vascular Ehlers-Danlos syndrome

**DOI:** 10.1371/journal.pone.0191220

**Published:** 2018-01-18

**Authors:** Nicola Chiarelli, Giulia Carini, Nicoletta Zoppi, Marco Ritelli, Marina Colombi

**Affiliations:** Department of Molecular and Translational Medicine, Division of Biology and Genetics, University of Brescia, Brescia, Italy; Ecole normale superieure de Lyon, FRANCE

## Abstract

Vascular Ehlers-Danlos syndrome (vEDS) is a dominantly inherited connective tissue disorder caused by mutations in the *COL3A1* gene that encodes type III collagen (COLLIII), which is the major expressed collagen in blood vessels and hollow organs. The majority of disease-causing variants in *COL3A1* are glycine substitutions and in-frame splice mutations in the triple helix domain that through a dominant negative effect are associated with the severe clinical spectrum potentially lethal of vEDS, characterized by fragility of soft connective tissues with arterial and organ ruptures.

To shed lights into molecular mechanisms underlying vEDS, we performed gene expression profiling in cultured skin fibroblasts from three patients with different structural *COL3A1* mutations. Transcriptome analysis revealed significant changes in the expression levels of several genes involved in maintenance of cell redox and endoplasmic reticulum (ER) homeostasis, COLLs folding and extracellular matrix (ECM) organization, formation of the proteasome complex, and cell cycle regulation. Protein analyses showed that aberrant COLLIII expression is associated with the disassembly of many structural ECM constituents, such as fibrillins, EMILINs, and elastin, as well as with the reduction of the proteoglycans perlecan, decorin, and versican, all playing an important role in the vascular system. Furthermore, the altered distribution of the ER marker protein disulfide isomerase PDI and the strong reduction of the COLLs-modifying enzyme FKBP22 are consistent with the disturbance of ER-related homeostasis and COLLs biosynthesis and post-translational modifications, indicated by microarray analysis. Our findings add new insights into the pathophysiology of this severe vascular disorder, since they provide a picture of the gene expression changes in vEDS skin fibroblasts and highlight that dominant negative mutations in *COL3A1* also affect post-translational modifications and deposition into the ECM of several structural proteins crucial to the integrity of soft connective tissues.

## Introduction

Vascular Ehlers-Danlos syndrome (vEDS, OMIM#130050) is a rare autosomal dominant inherited connective tissue disorder caused by mutations in the *COL3A1* gene encoding the pro-α 1 chain of type III procollagen (COLLIII) [1]. In the past, vEDS diagnosis was based on Villefranche criteria [2] and nowadays on their revision according to the 2017 international classification of EDS [3], which includes a family history with documented causative variant in *COL3A1*, a clinical history of arterial rupture, dissection or aneurysm, rupture of the large intestine, pregnancy complications at young ages, and carotid-cavernous fistula [4]. Vascular fragility and abnormal vessel structure cause easy bruising, severe varicosities, arterial dissection or aneurysm formation and eventual arterial or intestinal rupture that can lead to fatal consequences and a reduced life span of patients [4,5]. The frequency is estimated as 1/50,000–1/200,000 [4].

The predominant expression of COLLIII in the walls of blood vessels and in hollow organs explains why its reduction due to *COL3A1* mutations leads to increased bruising, arterial and bowel rupture, and uterine, cervical, and vaginal fragility during pregnancy and delivery [4]. vEDS patients also exhibit a constellation of clinical manifestations including thin translucent skin with visible venous pattern, spontaneous pneumothorax, acrogeria, congenital hip dislocation, hypermobility of small joints, tendon and muscle rupture, gingival recession and fragility, early onset varicose veins, and characteristic facial appearance [3,4].

Current treatment is symptomatic only, since no causal therapy exists. Surgical treatment of vascular disease in vEDS patients has been limited because of friable and fragile tissues [4]. A prospective study conducted in vEDS patients disclosed a preventive effect of celiprolol, a cardioselective β blocker with β2 agonist vasodilatory properties, which decreased the incidence of vascular complications [6].

COLLIII is a homotrimer formed by the binding of 3 α1(III) chains with a triple-helix structure in the central region. The amino acid sequence of the triple helical domain is characterized by repeated Gly-X-Y motifs that allow the correct folding of α-monomers. As observed in other COLLs-related diseases, i.e., Osteogenesis imperfecta, spondyloepiphyseal dysplasia, achondrogenesis, Stickler syndromes, Ullrich congenital muscular dystrophy, Bethlem myopathy, and other subtypes of EDS [3,7], mutations to glycine residues in *COL3A1* that disrupt the triple-helix motif constitute a frequent pathogenic mechanism. Indeed, *COL3A1* missense mutations altering the glycine codons together with in-frame splice mutations in the triple helical domain account for the majority of disease-causing variants and lead to misfolding of COLLIII in the endoplasmic reticulum (ER) and retention of seven eighths of misfolded procollagen trimers in the cell with consequent enlargement of ER vesicles [8–10]. This type of mutations produces a dominant negative effect on the protein, thereby inhibiting extracellular accumulation of mature COLLIII. Mutations leading to premature termination of translation and nonsense-mediated mRNA decay are rare (less than 5%). These null mutations cause half the normal amount of COLLIII to be secreted [11,12]. Some genotype-phenotype correlations have been recognized. Indeed, the nature of the specific *COL3A1* mutation influences life expectancy. Splice site mutations that lead to exon skipping have the lowest median survival. Substitutions of bulky residues (Arg, Asp, Glu, Val) for glycine residues in the triple helical domain are usually less severe than splice site mutations, but more consequential than substitutions by smaller residues (Ala, Ser, Cys). Heterozygosity for *COL3A1* null alleles typically produces a milder clinical phenotype with a significant delayed onset of complications [4, 9–12].

Although disruption of the COLLIII triple helical structure leads to abnormal protein folding, the molecular mechanism thereby contributing to the etiology of vEDS is poorly studied. Currently, there are two available mouse models for vEDS, both are homozygous or heterozygous nulls, which represent only about 5% or less of all known cases of vEDS patients [13–15]. While *Col3a1*^*+/−*^ mice present a very mild phenotype, as suggested by a low incidence of vascular events, homozygotes exhibit a severe perinatal mortality owing to arterial ruptures [13]. Cultured skin fibroblasts of vEDS patients synthesize aberrant COLLIII that is not organized into the extracellular matrix (ECM) as well as COLLI and fibronectin (FN) [16,17].

To gain insights into altered gene expression pattern and dysregulated biological processes underlying molecular pathology of vEDS, we carried out a transcriptome-wide expression profiling in cultured skin fibroblast strains derived from three vEDS patients with different structural mutations in *COL3A1*.

## Patients, materials and methods

### Clinical evaluation of vEDS patients

This study was approved by the local Ethical Committee “Comitato Etico di Brescia, ASST degli Spedali Civili, Brescia, Italia”, registration number NP2658 and performed in accordance with the Declaration of Helsinki Principles. Written informed consent was obtained from all patients and control donors to the study and for skin biopsy according to Italian bioethics laws. Subjects selected for this study were evaluated in the Centre of Heritable Connective tissue disorders and Ehlers-Danlos syndromes of the Spedali Civili of Brescia. vEDS patients were positive for the criteria of the Villefranche nosology [2] and the 2017 revised nosology [3]. In particular, the three vEDS patients included in this study were previously characterized for three different structural mutations in *COL3A1* [18]. vEDS patient 1 (P1) was heterozygous for the c.709G>A transition (p.Gly237Arg); patient 2 (P2) carried the c.951+6T>C splice site mutation that causes in-frame skipping of exon 14; patient 3 (P3) harbored the c.1835G>A missense variant (p.Gly612Asp). These patients have been previously reported as P.2, P.4, and P.10 in [18], respectively. A summary of their main clinical features is reported in **S1 Table**.

### Cell cultures and antibodies

Skin fibroblast cultures from three vEDS patients and nine unrelated age-matched healthy donors were established in our lab from skin biopsies by standard protocols. Fibroblasts were grown *in vitro* at 37° C in a 5% CO_2_ atmosphere in Earle’s Modified Eagle Medium (MEM) supplemented with 2 mM L-glutamine, 10% FBS, 100 μg/ml penicillin and streptomycin (Life Technologies, Carlsbad, CA, USA). Cells were expanded until full confluency and then harvested by 0.25% trypsin/0.02% EDTA treatment at the same passage number (from 3^rd^ to 6^th^).

Goat anti-COLLIII polyclonal antibody (Ab), and mouse anti-elastin (ELN) (clone 10B8) monoclonal antibody (mAb) were from Millipore-Chemicon Int. (Billerica, MA, USA). Anti-fibrillin (FBN) mAb (clone 11C1.3) was from NeoMarkers (Fremont, CA). Anti-EMILINs mAb was kindly provided by Prof. P. Bonaldo (University of Padova, Italy). The rabbit anti-versican core protein Ab was from Affinity Bioreagents (Golden, CO), the rabbit anti-perlecan core protein Ab and mouse anti-GAPDH mAb (clone 0411) were from Santa Cruz Biotec, Inc. (Heidelberg, Germany), the anti-decorin (DCN) mAb (clone 115402) was from R&D Systems, Inc. (Minneapolis, MN, USA), and the anti-heparan sulfate glycosaminoglycan chains (HS-GAGs) mAb was from USBiological Life Sciences (Swampscott, MA, USA). Rabbit anti-protein disulfide isomerase (PDI) Ab was from Novus Biologicals (Milano, Italy). Mouse anti-FK506-binding protein 22 (FKBP22) Ab was from Abnova (Walnut, CA, USA). Rhodamine-conjugated anti-goat IgG and anti-mouse IgM secondary Abs were from Calbiochem–Novabiochem INTL, and the Alexa Fluor 488 anti-rabbit and Alexa Fluor 594 anti-mouse secondary Abs were from Life Technologies. Horseradish peroxidase (HRP)-conjugated goat anti-mouse and anti-rabbit secondary Abs were from Sigma Chemicals (St. Louis, MO, USA).

### Immunofluorescence microscopy (IF)

To analyze the COLLIII-ECM organization, vEDS fibroblasts, grown for 48 h, were immunoreacted as previously reported [16]. In brief, cold methanol fixed fibroblasts were immunoreacted with 1:100 anti-COLLIII Ab. To analyze FBNs and ELN organization into ECM, cells were grown, fixed, and immunoreacted as reported in detail previously [19]. In particular, the FBNs organization was monitored 48 h after seeding: cold methanol fixed cells were reacted for 1 h with 1 μg/ml anti-FBNs mAb, which recognizes all FBN isoforms. The ELN organization was investigated 7 days after seeding, by fixing fibroblasts in 1% paraformaldehyde (PFA) for 20 min, treating 1 h at 37° C with 10 U/ml hyaluronidase diluted in MEM supplemented with 5% FBS, and immunoreacting for 1 h with 1:50 diluted anti-ELN mAb. EMILINs assembly was investigated 48 h after seeding by fixing cells in cold methanol and reacting for 75 min with 1 μg/ml anti-EMILINs mAb. The HS-GAGs, perlecan, DCN, and versican organization was analyzed 48 h from seeding by fixing cells with 3% PFA in PBS for 20 min. Subsequently, cells were incubated for 1 h with primary Abs (anti-HS-GAGs and anti-perlecan at 10 μg/ml; anti-DCN at 25 μg/ml; and anti-versican diluted 1:50 in BSA 1%). To analyze the distribution of ER marker PDI, fibroblasts grown for 48 h were fixed in cold methanol and immunoreacted for 2 h with 1:100 rabbit anti-PDI Ab. FKBP22 expression was analyzed by fixing 48 h-grown fibroblasts in 3% PFA/60 mM sucrose for 7 min, permeabilizing for 2 min in 0.5% (v/v) Triton X-100, blocking with 3% goat normal serum/PBS 1x for 1 h, and immunoreacting for 2 h with 1:100 anti-FKBP22 Ab. After washing in PBS, cells were incubated for 1 h with anti-mouse or anti-rabbit secondary Abs conjugated to Alexa Fluor 594 and 488, or with rhodamine-conjugated anti-goat IgG. IF signals were acquired by a CCD black-and-white TV camera (SensiCam-PCO Computer Optics GmbH, Germany) mounted on a Zeiss fluorescence Axiovert microscope and digitalized by Image Pro Plus software (Media Cybernetics, Silver Spring, MD, USA). All experiments were repeated three times.

### Western blotting (WB)

To analyze PDI and FKBP22, one control and three vEDS fibroblasts’ strains were cultured in complete MEM for 48 h. In particular, PDI was evaluated in cell extracts obtained by O.N. cell lysis and scraping at +4° C in buffer containing 20 mM Tris-HCl pH 6.8, 135 mM NaCl, 10% glycerol. 1% NP40, 1 μg/ml leupeptin, 4 μg/ml pepstatin, 0.1 UI/ml aprotinin, and centrifuged at 12,000 rpm at +4° C for 15 min. FKBP22 was analyzed after O.N. cell lysis and scraping at +4° C with buffer containing 50 mM Tris-HCl pH 7.4, 1% NP40, 0.5% DOC, 0.1% SDS, 150 mM NaCl, 2 mM EDTA, 1 mM PMSF, 1 μg/ml leupeptin, 4 μg/ml pepstatin, and centrifuged at 12,000 rpm at +4° C for 10 min. Protein concentration was evaluated using detergent compatible Bio-Rad D_c_ Protein Assay (Bio-Rad Laboratories, Hercules, CA, USA) and 30 μg of proteins were separated in reducing conditions by electrophoresis in 8% and 8%-12% SDS-PAGE, for PDI and FKBP22, respectively. After nitrocellulose sheet transfer, membranes were blocked O.N. at 37° C in 5% non-fat dry milk in TBS-0.1% Tween 20 (TBS-T), and immunoreacted at room temperature (RT) with 1:1,000 anti-PDI Ab diluted in TBS-T and 1:500 anti-FKBP22 Ab diluted in 5% dry milk/TBS-T. After washing in TBS-T, membranes were incubated for 2 h at RT with HRP-conjugated anti-rabbit and anti-mouse IgGs, respectively, diluted 1:1,000 in TBS-T and developed using ECL method (Pierce). After stripping, the two filters were reprobed at RT with 1:10,000 anti-GAPDH mAb diluted in TBS-T, as loading control, and with the anti-mouse secondary Ab, as reported above. The semi-quantitative evaluation of the Integrated Optical Density (IOD) of the bands was performed using the Image Pro Plus program (Media Cybernetics). The reported values are the means ± standard error of mean (SEM) of the ratios between the IOD of the sample and control protein bands detected in the same lane, obtained in three independent experiments. Statistical analysis of these data was determined by one-way ANOVA followed by Dunnett’s multiple comparisons post hoc test. Analyses were performed with GraphPad Prism software (GraphPad Software Inc., USA).

### Microarray procedures

Total RNA was extracted from skin fibroblasts of three patients and nine controls using the Qiagen RNeasy kit, according to manufacturer’s protocol (Qiagen, Hilden, Germany). RNA quality control was assessed on an Agilent 2100 BioAnalyzer (Agilent Technologies, Santa Clara, CA, USA). Transcriptome-wide expression profiling was performed using the Affymetrix Gene 1.0 ST platform. Microarray analysis was performed starting from 250 ng of total RNA per sample; labeled targets were prepared using Ambion Whole Transcript Expression Kit (Life Technologies) and GeneChip WT Terminal Labeling and Controls Kit (Affymetrix UK Ltd, Wycombe La High Wycombe, UK) in accordance with manufacturers’ instructions. Total RNA was primed with synthetic primers containing a T7 promoter sequence, reverse transcribed into first-strand cDNA and converted into double-stranded cDNA. Following the *in vitro* transcription, cRNA were reverse transcribed and the corresponding cDNA was fragmented, biotin labeled, and hybridized over night at 45° C onto the arrays. The chips were then washed in the Fluidics station FS 450, scanned using the scanner 3000 7G system, and analyzed with the Affymetrix GeneChip Operating Software. The resulting raw array data were analyzed using Partek Genomics Suite software (Partek Inc., St. Louis, MO, USA). Briefly, the microarray raw datasets were reprocessed by the background correction, normalization and summarization of probe intensities using the robust multiarray average analysis to determine the specific hybridizing signal for each probe set. After background correction, the expression data were corrected for perfect match intensity and were transformed in base-2 logarithm. Quality control was performed by investigating principal component analysis to detect grouping patterns in the samples and to identify the outliers and evaluate whether batch effect significantly affected the data. To detect differentially expressed genes (DEGs) between vEDS *vs* control cells, a one-way ANOVA analysis was used as a criterion for the selection of DEGs. To assess significant differences in the gene expression profile in vEDS fibroblasts, we selected the genes that had more than a 1.5-fold change and p-value <0.05. Multiple testing correction was applied to control the false-discovery rate (FDR) using the Benjamini–Hochberg (BH) procedure [20]. Genes with a FDR <0.05 were selected as differentially expressed and retained for further analysis. Database for Annotation, Visualization and Integrated Discovery (DAVID) v6.8 and ToppGene suite database were queried for identification of significantly enriched functional annotations, Gene Ontology (GO) biological process, and pathways analysis. Specifically, the main GO categories were examined with a FDR <20% after BH correction. All microarray data are MIAME compliant, and raw array data and processed data were deposited to the Gene Ontology Omnibus Database (GEO Accession Number GSE102042).

### Quantitative real-time PCR

Relative expression levels of a series of selected DEGs identified by array analysis were confirmed by quantitative real-time PCR (qPCR) using different RNA extractions obtained from skin fibroblast cultures of patients and healthy subjects. 3 μg of total RNA were reverse-transcribed with random primers by standard procedure. qPCR was performed with SYBR Green qPCR Master Mix (Life Technologies), 10 ng of cDNA, and with 10 μM of each primer set with the following cycling conditions: initial denaturation for 30 s at 95° C, followed by 40 cycles of 95° C for 15 s and 60° C for 60 s, in a ABI PRISM 7500 Real-Time PCR System. *HPRT*, *GAPDH*, *ATP5B*, and *CYC1* reference genes were amplified for normalization of cDNA loading. Amplification plots, dissociation curves, and threshold cycle values were generated by ABI Sequence detection system software version 1.3.1. The list of primers used for qPCR analyses is reported in **S2 Table**. Relative mRNA expression levels were normalized to the geometric mean of these reference genes and analyzed using the 2^-(ΔΔCt)^ method. Results were expressed as the mean value of relative quantification ± SEM. Statistical significance between groups was determined using unpaired Student’s t-test. Analyses were performed with GraphPad Prism software.

## Results

### Gene expression profiling

**Fig 1:**
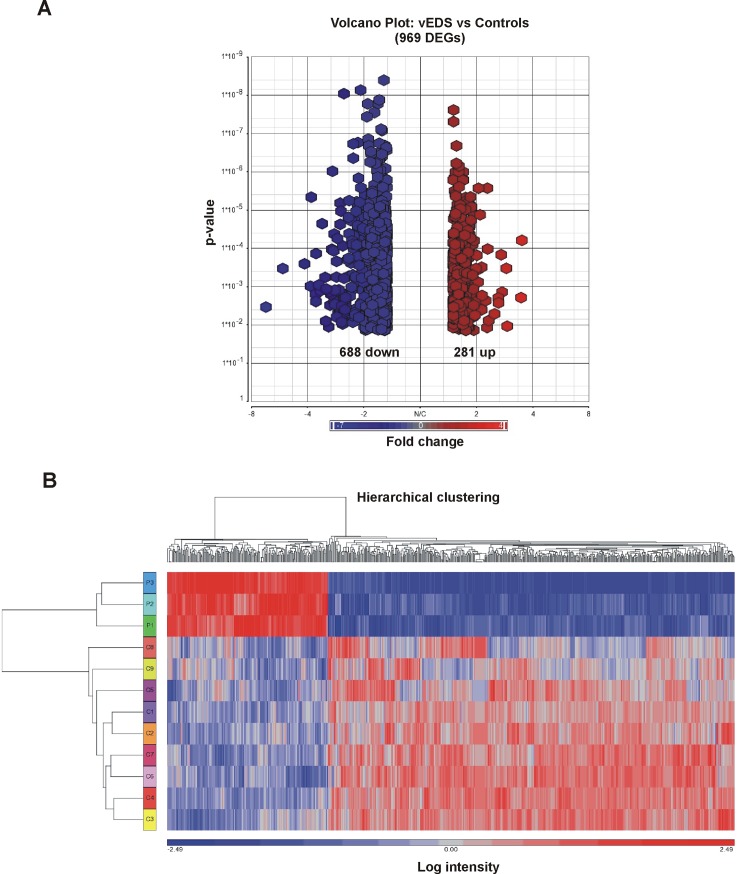
Volcano plot and hierarchical clustering analyses for differentially expressed genes in skin fibroblasts from vEDS patients and control subjects. **A**: The volcano plot depicts all statistically significant 969 DEGs (688 down-regulated, 281 up-regulated) identified in vEDS cells. The fold-change of DEGs on the x-axis *vs* the statistical significance (FDR-adjusted p-value <0.05) on the y-axis is shown; the up-regulated genes are reported in red, and the down-regulated genes are in blue. **B**: Hierarchical clustering analysis of DEGs identified in vEDS fibroblasts. Although fibroblasts from only 9 control subjects (C) and 3 vEDS patients (P) were analyzed, this analysis showed the presence of two distinct clusters of transcripts that clearly distinguish the patients from the controls. The red color represents high gene expression, and blue represents low gene expression.

To identify genes potentially involved in the molecular mechanisms associated with dominant negative *COL3A1* mutations, transcriptome-wide expression analysis was carried out comparing gene expression pattern between patients’ and controls’ skin fibroblasts. Gene expression profiling identified a total of 969 DEGs in vEDS patients’ cells when compared to controls: of these, 281 genes were up-regulated, and 688 were down-regulated (**S3 Table**). Panel A in **Fig 1** represents the volcano plot that illustrates the statistical significance (FDR) *vs* fold-change of DEGs, and **Table 1** reports a selection of the most up- and down-regulated DEGs (FDR < 0.05 and fold change threshold ≥ 1.5). To group transcripts with similar expression profiles between patients and controls, hierarchical clustering of DEGs was conducted (Panel B, **Fig 1**).

**Table 1:** Selection of DEGs identified in vEDS patients’ skin fibroblasts.

Gene symbol	Gene description	FDR 5%	Fold change
**Down-regulated genes**			
*FBN2*	Fibrillin 2	0,00342225	-6,68
*TNFAIP6*	Tumor necrosis factor, alpha-induced protein 6	0,000335479	-5,42
*PTCH2*	Patched 2	0,000254195	-4,15
*CCNB2*	Cyclin B2	0,000971685	-3,85
*HIST1H4L*	Histone cluster 1, H4l	4,60E-06	-3,82
*EDNRA*	Endothelin receptor type A	0,00157991	-3,62
*LINC01123*	Long intergenic non-protein coding RNA 1123	0,0024528	-3,60
*CPM*	Carboxypeptidase M	0,000137021	-3,60
*TOP2A*	Topoisomerase (DNA) II alpha	0,000900395	-3,48
*PBK*	PDZ binding kinase	0,00127233	-3,40
*ANLN*	Anillin actin binding protein	0,00125763	-3,38
*DDIT4*	DNA damage inducible transcript 4	2,26E-05	-3,34
*CENPK*	Centromere protein K	0,000570332	-3,22
*KIF20A*	Kinesin family member 20A	0,00792219	-3,19
*TPX2*	TPX2, microtubule-associated	0,00114946	-3,17
*DLGAP5*	Discs, large (Drosophila) homolog-associated protein 5	0,0112409	-3,08
*SHCBP1*	SHC SH2-domain binding protein 1	0,00135065	-3,04
*PCLAF*	PCNA clamp associated factor	0,00306261	-3,03
*PRC1*	Protein regulator of cytokinesis 1	0,00158038	-2,99
*CCNA2*	Cyclin A2	0,000104826	-2,96
*HIST1H2BM*	Histone cluster 1, H2bm	9,67E-07	-2,94
*LOXL3*	Lysyl oxidase-like 3	0,00011596	-2,90
*BUB1*	BUB1 mitotic checkpoint serine/threonine kinase	0,00287589	-2,88
*SKA3*	Spindle and kinetochore associated complex subunit 3	0,00123109	-2,85
*POSTN*	Periostin, osteoblast specific factor	0,00288956	-2,84
**Up-regulated genes**			
*TMEM130*	Transmembrane protein 130	6,15E-05	3,50
*MIR221*	MicroRNA 221	0,00194072	3,46
*ABCA9*	ATP binding cassette subfamily A member 9	0,0108267	2,92
*LRRFIP1*	Leucine rich repeat (in FLII) interacting protein 1	0,000332014	2,89
*EGR2*	Early growth response 2	0,00138662	2,74
*OCR1*	Ovarian cancer-related protein 1	0,000147134	2,71
*LOC441081*	POM121 membrane glycoprotein (rat) pseudogene	0,00265316	2,62
*HOXD11*	Homeobox D11	0,00196885	2,61
*LOC101929738*	Putative POM121-like protein 1	0,00512594	2,50
*JAM2*	Junctional adhesion molecule 2	0,00766174	2,32
*C9orf131*	Chromosome 9 open reading frame 131	0,00010363	2,29
*FHOD3*	Formin homology 2 domain containing 3	2,67E-06	2,28
*HSD17B6*	Hydroxysteroid (17-beta) dehydrogenase 6	0,0117282	2,28
*SDPR*	Serum deprivation response	0,00232078	2,24
*WISP2*	WNT1 inducible signaling pathway protein 2	0,00170072	2,14
*CDA*	Cytidine deaminase	0,00401697	2,13
*MIR218-2*	MicroRNA 218–2	0,000309256	2,13
*ND6*	NADH dehydrogenase, subunit 6 (complex I)	0,00196414	2,12
*RAPH1*	Ras association (RalGDS/AF-6) and pleckstrin homology domains 1	1,33E-05	2,09
*OR2L8*	Olfactory receptor, family 2, subfamily L, member 8	2,68E-06	2,05
*LIPN*	Lipase, family member N	0,00015225	2,01
*SLC4A4*	Solute carrier family 4 (sodium bicarbonate cotransporter)	0,000335655	1,98

To identify biological processes that were over- or under-represented in patients’ cells, we classified all up- and down-regulated genes according to the GO categories by using the DAVID and ToppGene biological databases. Only GO categories with FDR <20% in each annotation term were considered relevant.

The most enriched GO clusters of the 281 up-regulated DEGs (**Table 2**, and **S4 Table**) were associated with genes having olfactory receptor activity and with functions related to regulation of transcription and involved in the re-organization of actin cytoskeleton.

**Table 2:** DAVID functional annotation clustering of 281 up-regulated genes in vEDS patients’ skin fibroblasts.

Cluster	Enrichment Score	Category	Term	FDR <20%
**1**	**2.27**	INTERPRO	IPR000725:Olfactory receptor	0,00330
		GOTERM_MF_FAT	GO:0004984~olfactory receptor activity	0,00333
		GOTERM_BP_FAT	GO:0007608~sensory perception of smell	0,00370
**2**	**2.22**	GOTERM_BP_FAT	GO:0016481~negative regulation of transcription	0,00201
		GOTERM_BP_FAT	GO:0010629~negative regulation of gene expression	0,00429
		GOTERM_BP_FAT	GO:0010558~negative regulation of macromolecule biosynthetic process	0,00810
**3**	**2.04**	INTERPRO	IPR003104:Actin-binding FH2 and DRF autoregulatory	0,00801
		UP_SEQ_FEATURE	domain:FH2	0,00910
		INTERPRO	IPR015425:Actin-binding FH2	0,00918
		SMART	SM00498:FH2	0,00983

Functional analysis of 688 down-regulated genes yielded 28 different GO clusters (**Table 3**, and **S5 Table**). The most prominent group of down-regulated DEGs was implicated in cell cycle process at several steps, such as telomere organization, separation of sister chromatids, nucleosome assembly, and included cell division cycle protein genes (*CDC7*, *CDC26*, *CDC27*, *CDCA8*, *CDC5L*), and cyclin-dependent kinase genes (*CDK1*, *CDK2*, *CDK5RAP2*). Another group of significantly down-regulated genes encoded various histone proteins, i.e., *HIST1H4L*, *HIST1H4F*, *HIST1H3B*, *HIST1H1B*, *HIST1H3I*. A set of significantly GO enriched terms contained several kinesin family genes, such as *KIF14*, *KIFC1*, *KIF2C*, *KIF4A*, *KIF11*, *KIF15*, *KIF18A*, *KIF20A*, which play a pivotal role in cell movements and intracellular trafficking, including chromosome and centrosome positioning during mitosis. Also, processes implicated in DNA damage response and DNA mismatch repair were dysregulated in vEDS, as indicated by low mRNA levels of a number of associated genes, such as *MSH2*, *MSH6*, *RFC1*, *RFC2*, *RFC4*, *SSBP1*, *ARMT1*, *CLSPN*, *RAD21*, *FANCI*, *INTS7*, *NSMCE2*, *MGME1*, *FANCB*.

**Table 3:** Selection of DAVID functional annotation clustering of 688 down-regulated genes in vEDS patients’ skin fibroblasts.

Cluster	Enrichment Score	Category	Term	FDR <20%
**1**	**23.4**	UP_KEYWORDS	Mitosis	1,14E-27
**2**	**8.86**	GOTERM_BP_FAT	GO:0007062~sister chromatid cohesion	2,05E-08
**3**	**6.23**	GOTERM_BP_FAT	GO:0032200~telomere organization	3,97E-08
		GOTERM_BP_FAT	GO:0006335~DNA replication-dependent nucleosome assembly	4,41E-07
**4**	**4.87**	UP_KEYWORDS	DNA damage	3,83E-06
		GOTERM_BP_DIRECT	GO:0006281~DNA repair	0,000055
**5**	**4.86**	GOTERM_CC_DIRECT	GO:0005739~mitochondrion	0,00033
		GOTERM_CC_DIRECT	GO:0005759~mitochondrial matrix	0,020508
**6**	**4.42**	GOTERM_BP_DIRECT	GO:0034080~CENP-A containing nucleosome assembly	1,01E-09
**7**	**3.79**	KEGG_PATHWAY	hsa03010:Ribosome	0,000293
**8**	**3.02**	KEGG_PATHWAY	hsa03040:Spliceosome	7,92E-04
**9**	**2.86**	KEGG_PATHWAY	hsa03030:DNA replication	2,76E-05
**10**	**2.83**	INTERPRO	IPR003593:AAA+ ATPase domain	0,001385
**11**	**2.554**	GOTERM_MF_DIRECT	GO:0098641~cadherin binding involved in cell-cell adhesion	0,002066
**12**	**2.552**	GOTERM_BP_DIRECT	GO:0007018~microtubule-based movement	4,10E-04
		INTERPRO	IPR001752:Kinesin, motor domain	5,13E-04
		GOTERM_BP_DIRECT	GO:0006890~retrograde vesicle-mediated transport, Golgi to ER	0,021448
**13**	**2.34**	GOTERM_BP_DIRECT	GO:0043161~proteasome-mediated ubiquitin-dependent protein catabolic process	5,88E-04
		KEGG_PATHWAY	hsa03050:Proteasome	0,004173
**14**	**1.91**	GOTERM_BP_DIRECT	GO:0045454~cell redox homeostasis	0,001175
		INTERPRO	IPR013766:Thioredoxin domain	0,009429
**15**	**1.89**	GOTERM_CC_DIRECT	GO:0005681~spliceosomal complex	0,003396
**16**	**1.85**	GOTERM_BP_DIRECT	GO:0051439~regulation of ubiquitin-protein ligase activity involved in mitotic cell cycle	9,27E-05
		GOTERM_BP_DIRECT	GO:0006977~DNA damage response, signal transduction by p53 class mediator resulting in cell cycle arrest	0,018513
**17**	**1.82**	GOTERM_BP_DIRECT	GO:0000722~telomere maintenance via recombination	6,42E-04
		KEGG_PATHWAY	hsa03430:Mismatch repair	2.56E-04

We also found that GO categories related to “ribosome”, “rRNA processing”, and “structural constituent of ribosome” were significantly enriched with many down-regulated genes encoding both large and small ribosomal proteins essential for ribosome biogenesis (*RPL27A*, *MRPS5*, *MRPL30*, *MRPL21*, *MRPS18C*, *MRPL15*, *RPL23*, *RPS3A*, *RPL22*, *RPL9*, *RPL21*, *RPS12*, *RPS27A*). Similarly, transcriptome profiling revealed significant expression changes of many genes belonging to the spliceosome machinery, a large RNA–protein complex that catalyzes pre-mRNA splicing (**Table 3**, and **S5 Table**). Functional classification of DEGs also indicated in patients’ cells an aberrant expression of several small nucleolar RNAs (snoRNAs, **S3 Table**), which represent a class of non-coding RNAs (ncRNAs) that primarily mediate post-transcriptional modification of ribosomal RNAs.

Gene set enrichment analysis also indicated decreased mRNA levels of a range of genes, i.e., *PSMC5*, *PSMB6*, *RMND5A*, *PSMB1*, *PSMA6*, *PSMC3*, *PSMA4*, *PSMD2*, *BUB3*, *RPS27A*, *FBXO45*, *PSMD9*, involved in protein catabolism dependent by ubiquitin-proteasome pathway (**Table 3**, and **S5 Table)**. Among them, *PSMC5*, *PSMB6*, *PSMB1*, *PSMA6*, *PSMC3*, *PSMA4*, *PSMD2*, and *PSMD9* encode different catalytic and non-catalytic subunits of the proteasome, a multicatalytic proteinase complex that represents the central hub of protein degradation in eukaryotic cells.

Cell redox balance and homeostasis of protein folding into ER seem to be also perturbed in vEDS cells, as shown by reduced expression of many related genes, such as *KDELC1*, *KDELC2*, *FKBP14*, *GCLC*, *AIFM1*, *DLD*, *TXN*, *DNAJC10*, *PDIA6*, *PDIA5*, *TXNDC9*, *PDIA4* (**S5 Table**). Among them, we found low mRNA levels of *TXN*, *PDIA4*, *PDIA5*, and *PDIA6* encoding a class of ER-resident enzymes of the thioredoxin proteins superfamily that catalyze the formation and rearrangement of disulphide bonds during protein folding. Functional classification of DEGs also revealed significant expression changes of several members of DnaJ heat shock protein family, i.e., *DNAJB11*, *DNAJB7*, *DNAJC10*, *DNAJC24*, and *DNAJC3*, a class of chaperones facilitating protein folding and ER homeostasis (**S5 Table**).

### Pathways enrichment analysis

To identify differentially expressed pathways in vEDS fibroblasts, an enrichment analysis was carried out on all 969 DEGs by querying both ToppGene and DAVID databases and selecting a threshold of FDR-adjusted p-value <0.05. As reported in **S6 Table**, cell cycle was the most perturbed pathway in patients’ cells, as shown by a significant decreased expression of a number of cell cycle regulating genes. Pathways enrichment analysis also highlighted that DNA replication and mismatch repair processes were impaired in vEDS fibroblasts. This is indicated by low transcriptional levels of different related genes including primase subunit 1 (*PRIM1*), DNA polymerase epsilon 2 (*POLE2*), single stranded DNA binding protein 1 (*SSBP1*), members of replication factor C (*RFC1*, *RFC2*, *RFC4*), belonging to the mini-chromosome maintenance complex (*MCM3*, *MCM6*, *MCM7*), and to the DNA mismatch repair MutS family (*MSH2*, *MSH6*).

Similarly, enrichment analysis revealed a dysregulation of proteasome apparatus, as showed by an altered expression pattern of several genes encoding various proteasomal subunits. Moreover, ribosome and spliceosome pathways were also affected in patients’ fibroblasts (**S6 Table**). Concerning ribosome, vEDS cells showed an impairment of this ribonucleoprotein complex, as shown by low expression levels of many transcripts that encode both large and small subunits of nuclear ribosomal proteins such as *RPL21*, *RPL22*, *RPL23*, *RPL27A*, *RPL36A*, *RPL9*, *RPS12*, *RPS27A*, *RPS3A*. Spliceosome machinery was also disturbed as indicated by impaired expression of a subset of genes encoding members of heterogeneous nuclear ribonucleoproteins family, i.e., *HNRNPA1*, *HNRNPA1L2*, factors that enhance pre-mRNA splicing (*PRPF18*, *PRPF38A*, *PRPF4*, *SYF2*, *SLU7*), and belonging to nuclear ribonucleoprotein complex (*SNRPB*, *SNRPD3*).

### Validation of microarray data by qPCR

**Fig 2:**
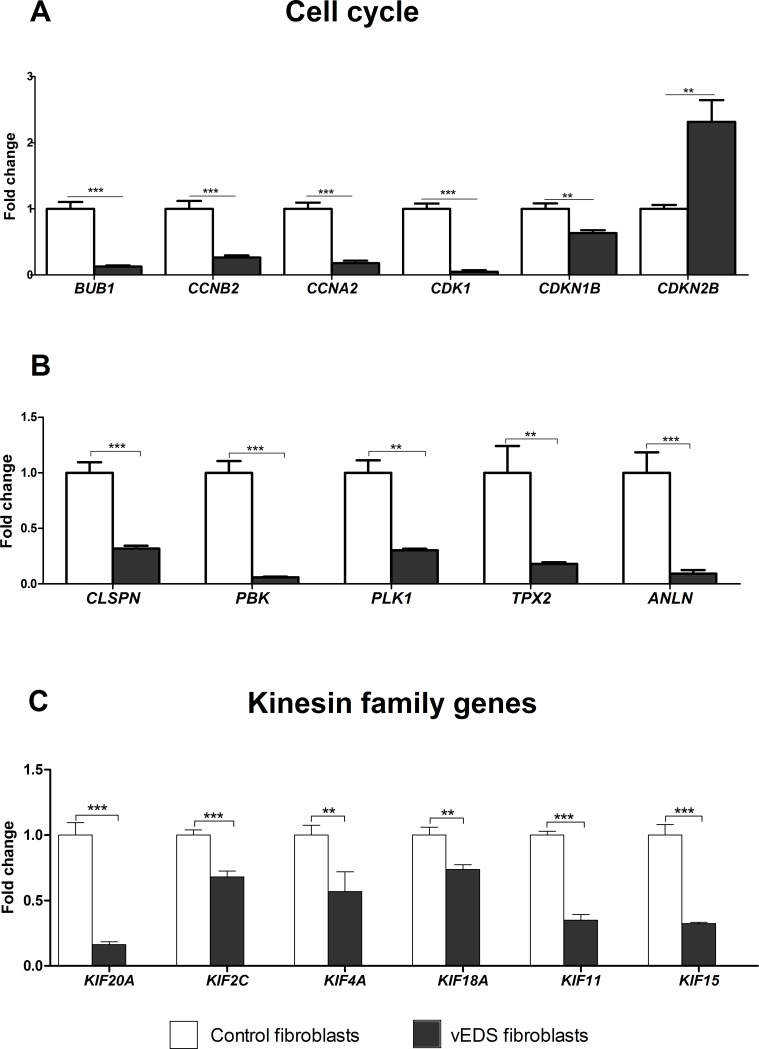
qPCR validation of genes involved in the cell cycle regulation. The relative mRNA expression levels of selected genes related to cell cycle (**A**, **B**), and genes belonging to kinesins family (**C**), were determined with the 2^-(ΔΔCt)^ method normalized with the geometric mean of different reference genes. Bars represent the mean ratio of target gene expression in three patients’ fibroblasts compared to five unrelated healthy individuals. qPCR was performed in triplicate, and the results are expressed as mean ± SEM. **p<0.01, and ***p<0.0001.

**Fig 3:**
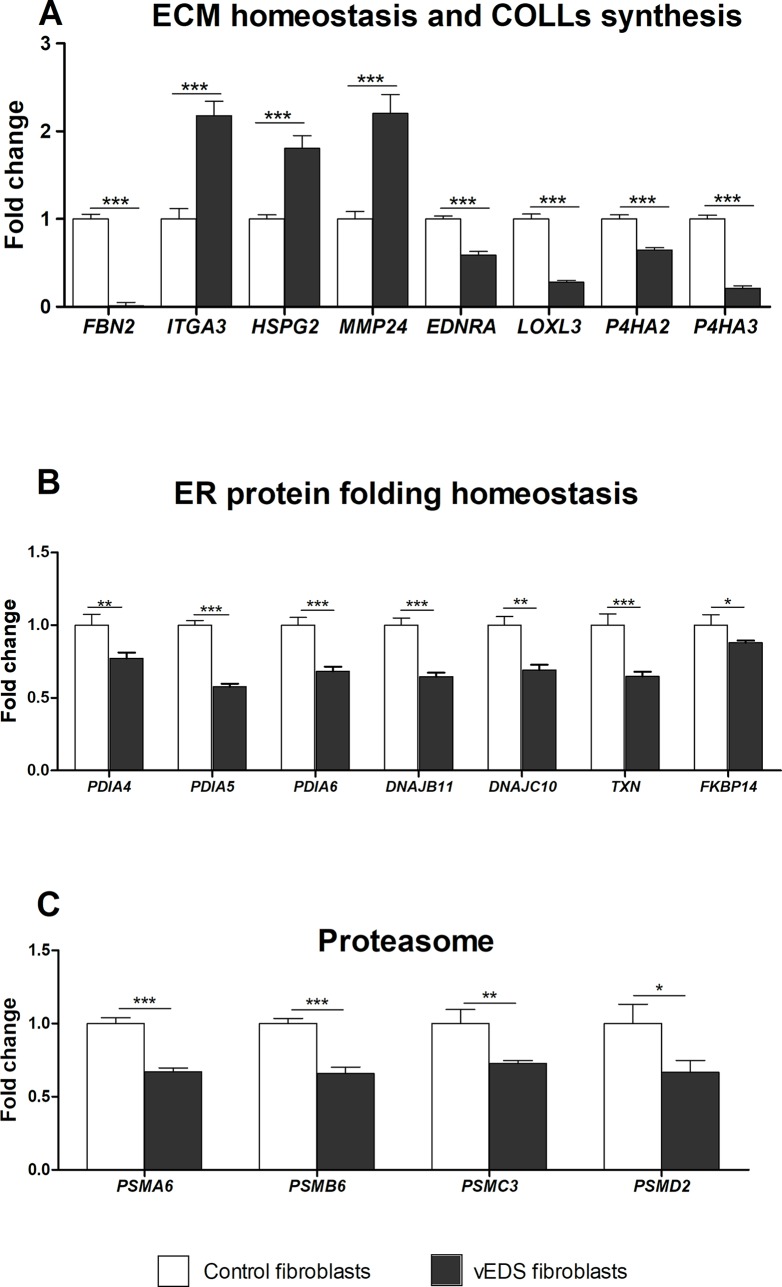
qPCR validation of genes involved in the maintenance of ECM architecture, cell redox and ER homeostasis, and belonging to proteasome complex. The relative mRNA expression levels of selected genes related to ECM structural integrity and COLLs synthesis (**A**), cell redox balance and ER homeostasis (**B**), and encoding different catalytic and non-catalytic subunits of the proteasome complex (**C**), were determined with the 2^-(ΔΔCt)^ method normalized with the geometric mean of different reference genes. Bars represent the mean ratio of target gene expression in three patients’ fibroblasts compared to five unrelated healthy individuals. qPCR was performed in triplicate, and the results are expressed as mean ± SEM. *p<0.05, **p<0.01, and ***p<0.001.

We validated the differential expression of a selection of DEGs by qPCR. Genes were prioritized based on their expression pattern and GO functional classification that was significantly perturbed in vEDS fibroblasts. Specifically, we focused on genes involved in cell cycle regulation (**Fig 2**), ECM organization and COLLs biosynthesis pathway, related to correct protein folding into ER, and belonging to the proteasomal complex (**Fig 3**). qPCR revealed a statistically significant decrease of a huge range of transcripts involved in the regulation of cell cycle, such as *BUB1*, *CCNA2*, *CCNB2*, *CDK1*, *CDKN1B*, *CLSPN*, *PBK*, *PLK1*, *TPX2*, *ANLN* (**Fig 2A and 2B**). Conversely, *CDKN2B*, which encodes a cyclin-dependent kinase (CDK) inhibitor, showed an enhanced expression in vEDS cells supporting microarray data (**Fig 2A**). qPCR also confirmed low transcriptional activity of various kinesin family genes, i.e., *KIF20A*, *KIF2C*, *KIF4A*, *KIF18A*, *KIF11*, and *KIF15* (**Fig 2C**). Significant changes of mRNA levels of several genes associated with ECM homeostasis and COLLs biosynthesis, such as *FBN2*, *ITGA3*, *HSPG2*, *MMP24*, *EDNRA*, *LOXL3*, *P4HA2*, *P4HA3*, were also confirmed (**Fig 3A**). We also verified the low transcription of many ER related genes, such as *PDIA4*, *PDIA5*, *PDIA6*, *DNAJB1*, *DNAJC10*, *TXN*, *FKBP14* (**Fig 3B**), as well as the low transcript levels of *PSMA6*, *PSMB6*, *PSMC3*, and *PSMD2*, which encode different catalytic and non-catalytic subunits of the proteasome complex (**Fig 3C**).

### Dominant negative mutations in *COL3A1* lead to the aberrant organization of several ECM structural components

**Fig 4:**
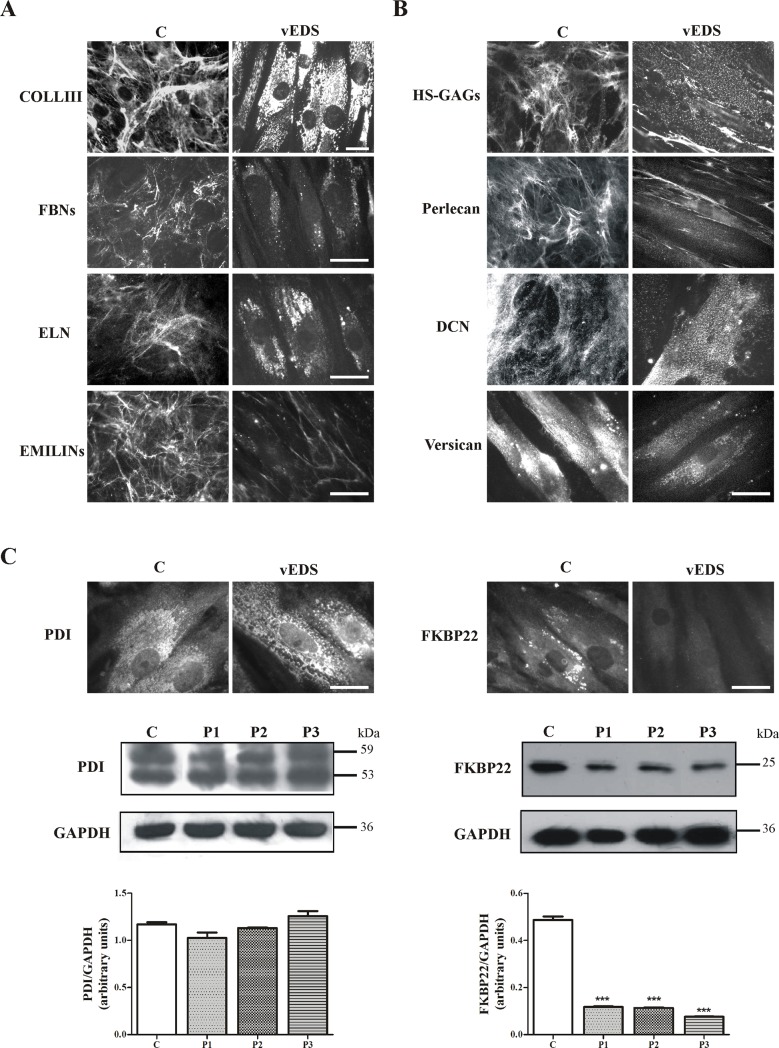
ECM organization and synthesis of ER-related enzymes in vEDS patients’ skin fibroblasts. (**A**) Control and patients’ cells were analyzed with specific Abs directed against COLLIII, FBNs, ELN, and EMILINs. EMILINs, FBNs, and ELN were investigated by labeling the cells with Abs recognizing all their isoforms. Scale bar: 10 μm. Images are representative of 2 control and 3 vEDS analyzed fibroblasts. (**B**) Control and vEDS fibroblasts were analyzed with specific Abs directed against HS-GAG chains, and the core proteins of the PGs perlecan, DCN, and versican. Scale bar: 10 μm. Images are representative of 2 control and 3 vEDS fibroblasts. (**C**) Control and vEDS cells were analyzed with specific Abs recognizing the ER marker PDI and the COLLs-modifying enzyme FKBP22. Scale bar: 10 μm. WB of 30 μg of proteins recovered from control (C) and three vEDS cell strains (P1, P2, P3), immunoreacted with the rabbit anti-human PDI and mouse anti-human FKBP22 Abs, detecting two 59 and 53 kDa bands and a band at 25 kDa, respectively. Loading control: GAPDH. Experiments were repeated three times. Representative images are shown. Protein bands were quantified by image analysis as described in Materials and Methods section. *** p <0.0001.

In vEDS patients’ skin fibroblasts, we previously demonstrated an abnormal synthesis and deposition of COLLIII into the ECM together with the disarray of FN and the lack of COLLs- and FN-specific integrin receptors [16,17]. IF analyses of COLLIII in the present skin fibroblasts with different structural mutations confirmed the ECM disorganization and intracellular retention of the aberrant protein (**Fig 4A**). The consequence of aberrant COLLIII on the organization, expression, and distribution of other structural ECM components, i.e., FBNs, ELN, EMILINs, HS-GAG chains, and the core proteins of the proteoglycans (PGs) perlecan, versican, and DCN was analyzed by IF. As shown in **Fig 4A**, FBNs were organized in fibrillar networks covering control fibroblasts, whereas these proteins were not assembled into the ECM of vEDS cells that showed rare and sparse cytoplasmic spots. This finding is in line with the strong down-regulation of the *FBN2* transcript shown by microarray analysis and confirmed by qPCR. Similarly, ELN was not organized into the ECM of 7 days-grown vEDS fibroblasts, in which the protein was detectable only in the cytoplasm. In addition, vEDS fibroblasts were also unable to produce a fibrillar well-organized ECM network of EMILINs, since only few fibrils were present in intercellular spaces. Moreover, control cells organized HS-GAGs in a rich fibrillar network, whereas in vEDS cells only few fibrils, localized in the extracellular spaces and discrete spots associated with the cell surface, were identified (**Fig 4B**). The HS-specific perlecan, organized by control fibroblasts in a fibrillar ECM meshwork and resembling the HS-GAGs distribution, was highly reduced in patients' fibroblasts. Likewise, the PGs versican and DCN showed a strong reduction of their expression in vEDS cells compared to control cells (**Fig 4B**).

Based on microarray results, the synthesis and organization of the two ER-related proteins, i.e., PDI and FKBP22, were investigated by IF and WB. Control and vEDS fibroblasts showed comparable levels of PDI, although in mutant cells the IF signals suggested an enlargement of ER vesicles (**Fig 4C**). IF of FKBP22 suggested lower cytoplasmic levels of this COLLs-modifying enzyme in patients compared to control cells; WB confirmed this down-regulation demonstrating a 4.1 to 6.4-fold decrease.

## Discussion

The present study reports the first molecular evidence of significant gene expression changes in vEDS cells that could provide insights into the pathogenesis of the disease. Previous studies showed that vascular disease in vEDS is not merely due to tissue fragility through quantification in patients’ plasma of increased levels of several vascular inflammatory mediators including TGFβ1 and TGFβ2 [21,22], as well as a high prevalence of platelet dysfunctions and low vitamin D serum concentration [5].

We recognize the limited number of analyzed samples, as well as it is not obvious that other ECM producing cells, i.e., vascular smooth muscle cells and arterial fibroblasts, could show the same results in terms of gene expression profile. However, skin fibroblasts have been historically used to study vEDS pathophysiology, since the disease is characterized by a strong cutaneous involvement, mimicking the vascular tissue fragility. In addition, since our patients harbored different structural *COL3A1* mutations, i.e., glycine substitution and in-frame exon skipping, which correspond to the most frequent type of the disease-causing variants, the present results should be representative of the majority of vEDS individuals. Besides, the present patients exhibited a full-blown expression of the disease with several major arterial events, in line with the limited genotype-phenotype correlation that associates this type of structural mutations with a more severe clinical phenotype [4,9–12]. In line with this assumption, our previous and current protein findings on patients’ fibroblasts indicated that dominant negative effect of structural *COL3A1* mutations is not limited to its homotrimers formation but also affects the structural integrity of different components crucial to the maintenance of soft connective tissues architecture. Structural misfolding mutations in COLLs-encoding genes or in other ECM-related molecules, such as *COL1A1*, *COL1A2*, *COL2A1*, *COL6A1*, *COL6A2*, *COL6A3*, and *COMP*, are well known to cause a dominant negative effect, leading to partial or complete cellular retention and/or degradation of mutant proteins, and normal proteins being assembled into mutant-containing multimers. This results in a severe protein deficiency as well as more general metabolic consequences and, if the mutant abnormally folded protein is secreted, in a further deleterious effect on ECM stability, architecture, and function through compromising the interactions with other ECM ligands [7]. About this, it is well established that structural alterations of the COLLI, associated with a certain degree of genotype-phenotype correlation, may exert a detrimental effect after chain assembly and helix folding, including the disruption of fibril formation, cell–cell and cell–matrix interactions, and the binding to several noncollagenous molecules in the ECM through its major ligand binding regions (MLBRs) [23]. More recently, the definition of the human COLLIII interactome revealed a nearly identical structural arrangement and functional domains as for COLLI, including cell interaction, fibrillogenesis and enzyme cleavage domains, intermolecular crosslink sites, and several MLBRs for different ECM components, such as other COLLs, PGs, and FN [24]. We previously showed that in vEDS skin fibroblasts the defective synthesis of COLLIII did not interfere with the COLLV secretion and its extracellular organization, but altered the deposition and proper assembly into the ECM of COLLI, which was mainly retained in the cytoplasm [16]. This finding is consistent with the known regulatory role of COLLIII in either the synthesis or deposition of heterotypic fibrils that largely contain COLLI [23,24]. In the present study, we demonstrated that the abnormal folding, secretion, and ECM organization of COLLIII cause an altered deposition and the disassembly of other structural constituents, such as FN, FBNs, ELN, and EMILINs [16,25], which provide a scaffold for elastogenesis and represent the major components of elastic fibers in elastic tissues including skin and arterial walls, and are essential to maintain the vascular ECM homeostasis [26].

Transcriptome profiling revealed the dysregulated expression pattern of a range of genes and related processes mainly involved in ECM organization/COLLs biosynthesis, maintenance of ER homeostasis, cell cycle regulation, and RNA processing.

### ECM organization/COLLs biosynthesis

Our data revealed significant expression changes of a subset of genes involved in the maintenance of ECM structural integrity and in the biosynthesis and post-translational modifications (PTMs) of COLLs and ELN, including *FBN2*, *HSPG2*, *EDNRA*, *LOXL3*, *P4HA2*, *P4HA3*, and *FKBP14*. *FBN2* is the most down-regulated transcript in vEDS fibroblasts and encodes fibrillin-2 that is a member of fibrillins, which are large extracellular macromolecules that polymerize to form the backbone structure of connective tissue microfibrils [27]. Fibrillin microfibrils are ECM structures with essential functions in the development and the organization of several tissues including blood vessels, and they can bind a number of other extracellular proteins, including latent TGFβ binding proteins (LTBPs), fibulins, and laminins [28]. Fibrillins interact closely with tropoelastin and integrins, and provide a scaffold for elastogenesis in elastic tissues [26]. Besides, fibrillin-2 interacts directly with fibrillin-1, EMILIN-1, and LTBPs to regulate the bioavailability of TGFβ, a growth factor and key regulator of connective tissue organization and blood vessels development and maintenance [27,29].

Our protein data showed a generalized disassembly of all isotypes of fibrillin in vEDS fibroblasts’ ECM, concomitant to the disorganization of EMILINs and ELN networks, providing an *in vitro* picture consistent with the pathological conditions of arterial walls reported in vEDS patients. Based on these protein findings, it might be reasonable to speculate a possible contribute of the TGFβ signaling in the molecular mechanisms of vEDS, as showed for different connective tissue disorders with cardiovascular complications, i.e., Marfan syndrome, Loyes-Dietz syndrome, and arterial tortuosity syndrome [19,30]. Nevertheless, a previous study on vEDS skin fibroblasts did not disclose aberrant canonical and noncanonical TGFβ pathways, despite the increased levels of TGFβ2 ligand in the cells’ media [22]. Anyway, in other ECM-producing cell types, such as smooth muscle cells, the involvement of TGFβ pathway cannot be excluded, therefore, more research is warranted to establish its possible role in the pathogenesis of vEDS.

*HSPG2* encodes perlecan, a PG present in the basement membranes, which binds to and cross-links many ECM components and cell-surface proteins, such as COLLI, COLLIV, laminin, nidogen, and FN [31]. It is also a key component of the vascular ECM with endothelial barrier function, and it is a potent inhibitor of smooth muscle cell proliferation, thus contributing to maintain vascular homeostasis [32]. Our protein data indicated the disorganization of the core proteins of PGs perlecan, versican, and DCN, which enhance COLL fibril formation [26], and their abnormal organization could contribute to the defective COLLIII fibril assembly in vEDS cells, as well as to the defective fibrillogenesis of other COLLs previously described in skin fibroblasts of different EDS types and in other connective tissue disorders with vascular involvement such as arterial tortuosity syndrome [16,17,19,33,34]. *EDNRA* encodes the endothelin receptor type A (ET-A) that interacts with receptor type B to mediate endothelin-1 (ET-1) signaling, a potent endothelium-derived vasoconstrictor peptide that can activate vascular smooth muscle cells, inducing proliferation, and synthesis of ECM proteins [35]. ET-1 causes vasoconstriction mainly via ET-A, which represents the principle subtype in the vascular medial layer of large arteries [36].

Biosynthesis of mature COLL fibrils is a multistep process spanning both intracellular and extracellular PTMs mediated by various enzymes and molecular chaperones that provide specific modifications such as hydroxylation of prolyl and lysyl residues, which are a prerequisite for correct protein folding [7]. In line with this, our expression data showed that *COL3A1* structural mutations are associated with the alteration of transcript levels of different COLLs-modifying enzymes, such as *LOXL3*, *P4HA2*, *P4HA3*, and *FKBP14*. *LOXL3* encodes a copper-dependent amine oxidase required for the formation of lysine-derived cross-links of COLLs and ELN fibrils [37]. The down-regulated *LOXL3* expression in vEDS fibroblasts and the concomitant COLLIII- and ELN-ECM disorganization suggest that in dermal fibroblasts collagenogenesis and elastogenesis should be co-regulated, according to Aziz and colleagues’ findings [38]. *P4HA2* and *P4HA3* encode two of the three isoforms of the α-subunit of collagen prolyl-4-hydroxylase, a tetrameric enzyme resident in the ER lumen that hydroxylates prolines on the procollagen peptide and serves as a chaperone for procollagen [39]. Hydroxylation of COLLs prolyl residues is essential for proper assembly and thermal stabilization of COLLs triple helices through intramolecular hydrogen bonding [40], since under-hydroxylation results in abnormal assembly, and reduced thermal stability of newly synthesized procollagen chains [39]. *FKBP14* encodes FKBP22, a peptidyl-prolyl cis-trans isomerase localized in the ER lumen [41] that interacts with different types of COLLs, i.e., COLLIII, COLLVI, and COLLX, and during COLLs triple helix formation catalyzes cis–trans-isomerization of peptide bonds, promoting in particular the COLLIII folding [39,42]. Biallelic mutations in *FKBP14* cause kyphoscoliotic EDS (kEDS-*FKBP14*) that is complicated by life-threatening cardiovascular complaints. Indeed, aortic rupture, internal carotid artery and celiac artery dissections, and hypogastric artery pseudoaneurysm rupture were reported [33,43–45]. In FKBP22-deficient skin fibroblasts a disturbed distribution and assembly of several ECM proteins, such as COLLI, COLLIII, COLLV, COLLVI, FN, tenascins, and thrombospondin has been described, suggesting that FKBP22 deficiency influences the proper deposition of different structural constituents of the ECM [33]. Moreover, the ER cisternae of FKBP22-deficient fibroblasts were dilated and filled of proteins [33]. The strong retention of COLLIII in vEDS fibroblasts’ cytoplasm here reported suggests a role of FKBP22 in the biosynthesis and secretion mechanism of this COLL, and the reduction of this enzyme may represent a common trait of *in vitro* cultured fibroblasts derived from individuals with kEDS and vEDS. In particular, both the *FKBP14* inactivation in kEDS-*FKBP14* cells and the reduction of FKBP22 in response to the misfolded COLLIII retention in ER of vEDS fibroblasts have a common downstream negative effect on the assembly of several structural proteins into the ECM. Indeed, enhanced storages of FN [25] and ELN (this work) in cytoplasmic compartments of vEDS fibroblasts were described, according to the notion that structural mutations in genes encoding for different COLLs, i.e., COLLI, COLLIII, and COLLV, affect not only the assembly but also the secretion pathways of other ECM components in EDS fibroblasts [16,17,33]. Furthermore, a role of FKBP22 as chaperone for one or more ECM proteins in vEDS cells such as in other cell types affected by abnormal COLLs secretion cannot be excluded.

### ER protein folding homeostasis

Besides *FKBP14*, transcriptome profiling also underlined the reduced expression of other genes with functions related to the maintenance of the ER protein folding homeostasis. ER not only provides mechanisms to facilitate proper folding of newly synthesized secretory and membrane proteins, but also harbors molecular machineries that eliminate proteins that fail to fold or assemble correctly [46]. Disturbance of ER redox state, as a consequence of oxidative stress, alteration of calcium homeostasis, or dysregulated chaperones activity, may result in un/misfolded, or aggregated proteins that will be targeted to degradation pathways or accumulate in cells [47]. About this, vEDS fibroblasts showed decreased mRNA levels of several members of DnaJ heat shock protein family, which are molecular co-chaperones that act as ER reductases by catalyzing the removal of non-native disulfides, enhancing protein folding [48]. Likewise, microarray data highlighted a diminished transcriptional activity of *TXN*, *PDIA4*, *PDIA5*, and *PDIA6* belonging to the thioredoxin superfamily that facilitate correct oxidative protein folding, thus ensuring ER-associated proteostasis [49]. Moreover, *PDIA4*, *PDIA5*, and *PDIA6* are members of an expanding proteins family known as protein disulfide isomerases that may also act as effectors of protein translocation of terminally un/misfolded proteins to the cytosol for their proteasomal degradation [50]. In this regard, we found down-regulation of many members of the proteasome complex, a proteolytic machinery that participates in the maintenance of protein homeostasis and its dysfunction contributes to the accumulation of protein aggregates [51]. Although in vEDS cells the protein levels of the ER marker PDI were not significantly different from control fibroblasts, its distribution seems to suggest an enlargement of ER vesicles, which should entail an accumulation of misfolded proteins, including COLLIII, and ensuing impairment of protein folding quality control. Alterations of ER redox state may also influence the cell death machinery by inducing caspase-dependent apoptosis through the down-regulation of Bcl-2 protein, which physiologically blocks the apoptotic cell death [52]. In line with these observations, our previous study demonstrated in vEDS fibroblasts the decrease of Bcl-2 protein expression and the activation of caspase enzymes, strongly supporting the propensity of these cells to undergo apoptosis [17].

Overall our molecular findings may provide further support to early evidences suggesting that the disturbance of ER related homeostasis could be likely associated with the disease mechanism as a result of abnormal protein folding with ensuing intracellular retention of mutant COLLIII chains and ER dilation [8,53–55]. Indeed, the ER disturbance could also be related to the down-regulation of the assembly of GAG chains and downstream organization of PGs, as shown by the reduction of perlecan, DCN, and versican in vEDS fibroblasts’ ECM. Moreover, increasing observations indicate ER stress as a mechanism involved in the pathogenesis of a range of systemic diseases including connective tissue disorders [56–59]. Despite this, our data did not reveal an enhanced expression of key regulators of unfolded protein response (UPR), an adaptive pathway to restore protein folding equilibrium [51]. However, the lack of a classical UPR activation (e.g., elevated BiP levels) was previously reported in skin fibroblasts of Osteogenesis imperfecta patients with mutations in triple helical domain of *COL1A1* [60], as well as in embryonic fibroblasts from a Marfan syndrome mouse model [61] and in patients’ fibroblasts with different types of mucopolysaccharidoses [62]. Therefore, it seems reasonable to presume that the nature of accumulated substrate, the tissue and mutation type can determine whether the UPR pathway will be triggered or not. Besides, it is likely that procollagen misfolding caused by mutations within the triple helix domain does not activate UPR [60,63], but rather leads to the accumulation of insoluble procollagen aggregates that could be eliminated by autophagy [64]. Additional functional work is required to verify whether these stress pathways are also activated in vEDS patients’ cells.

### Cell cycle

The most down-regulated genes in vEDS patients ‘cells were implicated in processes related to cell division, DNA replication, telomere organization, and associated with nucleosome and chromatin assembly. We also found significant expression changes of a large range of transcripts encoding histone proteins that could reflect a diminished DNA synthesis, given that histones enter in nucleosome formation and proper DNA wrapping after the S phase of cell cycle [65]. Another subset of markedly down-regulated transcripts encodes several kinesin family members, a class of motor proteins involved in cell movements and intracellular vesicles’ trafficking from ER, and in chromosome and centrosome positioning during mitosis [66]. DNA repair and cell cycle were the most down-regulated pathways in vEDS cells. These processes are commonly damaged in primary human fibroblast strains undergoing a cellular aging process as a consequence of finite proliferative capacity or oxidative stress [67]. The β-galactosidase assay performed on vEDS fibroblasts indicated that these cells do not activate precocious aging mechanism (data not shown), but they undergo an ECM-deficiency-mediated pre-apoptotic state, in which caspases’ activity is up-regulated according to the down-expression of Bcl-2 [17]. It is well known that cell cycle progression in eukaryotic cells is strictly regulated by both integrin-mediated adhesion to the ECM and by binding of growth factors to their receptors [68]. The rescue from *anoikis* is driven in vEDS fibroblasts by a survival signaling transduced through the cross-talk between the αvβ3 integrin and EGF receptor [17]. Recently, an anti-apoptotic role of FKBP22, favoring cell proliferation, by the over-expression of Bcl-2 and the silencing of caspases has been reported. Conversely, the shRNA-mediated *FKBP14* inhibition induces cell cycle arrest and apoptosis [69]. In vEDS fibroblasts, the role of FKBP22 in the pre-apoptotic condition needs further examination.

Also, in patients’ fibroblasts the DNA damage response and the cyclins/CDKs/CDK inhibitors machine seem to be perturbed. In particular, the up-regulation of *CDKN2B*, encoding the p15 protein that is an inhibitor of the cyclin D/CDK4 and CDK6 complexes, suggests a possible delay of G1 phase transition to overcome DNA damages and stress conditions, whereas the down-regulation of *CDKN1B* transcription, which is an inhibitor of cyclin E/CDK2 complex, suggests an opposite response of cells to overcome apoptosis’ activation and go on cell cycle. This cell cycle phases’ perturbation could be a consequence of accumulated DNA mismatches, probably not repaired by not-functioning mismatch repair mechanism. *COL3A1* mismatches can be responsible of misfolded COLLIII trimers, which are not processed by proteasome and accumulate in the ER enlarged cisternae blocking vEDS cells’ trafficking. Gene expression profiling also revealed an aberrant expression of many genes implicated in pathways that regulate RNA processing, spliceosome and ribosome assembly. Among these DEGs, vEDS cells showed a differential expression of snoRNAs, a class of ncRNAs involved in the ribosomal RNA maturation and biogenesis [70]. Accumulating evidence suggested that various types of ncRNAs including snoRNAs can also participate in the regulation of gene expression, cell cycle, DNA repair, genome integrity, as well as in human diseases [71,72]. However, clarifying the mechanism responsible for the down-regulation of DNA repair and cell cycle associated pathways, and whether altered snoRNAs expression may affect transcriptional regulation of different cell cycle-related genes or those involved in RNA processing and ribosome biogenesis, need further experimentation.

In conclusion, our approach illustrates global mRNA profiling changes of several genes and related biological processes that could offer novel insights into the molecular pathology of vEDS. Protein data revealed that dominant negative effect triggered by COLLIII misfolding also affects the organization of structural proteins crucial to the integrity of soft connective tissues. Further studies are needed to delineate in detail the functional role of the identified gene expression changes in vEDS fibroblasts and to identify distinct molecular signature(s) that may contribute to the mechanistic events that cause vascular wall weakness of this severe connective tissue disorder.

## Supporting information

S1 Table Summary of the main clinical features of vEDS patients. (DOCX)

S2 Table List of primers used for qPCR analyses. (DOCX)

S3 Table Full list of DEGs identified in vEDS patients’ skin fibroblasts. (XLSX)

S4 Table DAVID functional annotation clustering of 281 up-regulated DEGs in vEDS patients’ skin fibroblasts. (XLSX)

S5 Table DAVID functional annotation clustering of 688 down-regulated DEGs in vEDS patients’ skin fibroblasts. (XLSX)

S6 Table Top canonical pathways perturbed in vEDS patients’ skin fibroblasts. (DOCX)
