# Supplementary material for: Transcriptome analysis of skin fibroblasts with dominant negative COL3A1 mutations provides molecular insights into the etiopathology of vascular Ehlers-Danlos syndrome
Source: PLoS One. 2018 Jan 18;13(1):e0191220. doi: 10.1371/journal.pone.0191220 (PMC5773204; doi:10.1371/journal.pone.0191220)
Supplement: S1 Table — (DOCX) [file pone.0191220.s001.docx]

**S1 Table. Summary of the main clinical features of vEDS patients.**

|  | **Patient 1 (P1)** | **Patient 2 (P2)** | **Patient (P3)** |
| --- | --- | --- | --- |
| **Sex** | M | F | M |
| **Age (years)** | 64 | 55 | 54 |
| **Age at diagnosis (years)** | 53 | 45 | 42 |
| **Mutation/Position** | c.709G>A/Ex 9 | c.951+6T>C/Int 14 | c.1835G>A/Ex 26 |
| **Effect** | p.(Gly237Arg) | In-frame ex14 skipping | p.(Gly612Asp) |
| **Family history** | + | + | + |
| **1^st^ complication (age/type)** | 33/synchronic hepatic and  splenic arteries rupture | 30/ileopsoas artery rupture | 41/hepatic artery dissection |
| **Arterial/Intestinal/Uterine fragility or rupture** | + | + | + |
| **Arterial rupture** | + | + | + |
| **Sigmoid colon perforation** | + | - | - |
| **Bruising** | - | + | + |
| **Thin, translucent skin** | + | + | + |
| **Characteristic facies** | + | + | + |
| **Hypermobility of small joints** | + | + | + |
| **Tendon and muscle rupture** | - | + | - |
| **Gingival recession/fragility** | + | + | NA |
| **Early onset of varicose veins** | - | + | - |
| **Other clinical features** | Aneurysms of renal, ICA,  and femoral arteries | Sp, aneurysms of femoral, iliac, and popliteal arteries | Intracranial and renal artery aneurysms |

+ present; -, absent; Sp, spontaneous splenic rupture; ICA, internal carotid artery; NA, not assessed. DNA mutation numbering is based on the cDNA sequence, and the +1 position corresponds to the A of the ATG translation initiation codon in the reference sequence (*COL3A1*: cDNA: NM_000090.3, protein NP_000081.1).
