# Supplementary material for: Transcriptome analysis of skin fibroblasts with dominant negative COL3A1 mutations provides molecular insights into the etiopathology of vascular Ehlers-Danlos syndrome
Source: PLoS One. 2018 Jan 18;13(1):e0191220. doi: 10.1371/journal.pone.0191220 (PMC5773204; doi:10.1371/journal.pone.0191220)
Supplement: S2 Table — (DOCX) [file pone.0191220.s002.docx]

**S2 Table. List of primers used for qPCR analyses.**

| **Gene** | **NCBI RefSeq** | **Amplicon size (bp)** | **Primer forward** | **Primer reverse** |
| --- | --- | --- | --- | --- |
| *ANLN* | NM_018685.4 | 93 | TCCAAAGCGACTCCTCACAT | CTGGTTCGCACAGCACTAAG |
| **ATP5B*** | NM_001686.3 | 127 | CCAATTCTAAATGCCCTGGA | GGCCTCTAACCAAGCCTTCT |
| *BUB1* | NM_004336.4 | 149 | CCTTTGGAGAACGCTCTGTC | TGTGAAGTCTCCTGGGCTCT |
| *CCNA2* | NM_001237.3 | 111 | CCTGCAAACTGCAAAGTTGA | AAAGGCAGCTCCAGCAATAA |
| *CCNB2* | NM_004701.3 | 106 | ACTGCTCTGCTCTTGGCTTC | TTTCTCGGATTTGGGAACTG |
| *CDK1* | NM_001786.4 | 135 | TACAGGTCAAGTGGTAGCCA | AGCACATCCTGAAGACTGACT |
| *CDKN1B* | NM_004064.4 | 112 | CGCAGGAATAAGGAAGCGAC | CTCCACAGAACCGGCATTTG |
| *CDKN2B* | NM_004936.3 | 90 | GCGAGGAGAACAAGGGCA | GTCGCACCTTCTCCACTAGT |
| *CLSPN* | NM_022111.3 | 100 | GAGGTGGGTTCTGAGGTTCA | TTTCATAGCTGCCCTGTCCA |
| **CYC1*** | NM_001916.3 | 140 | CACGGAGGATGAAGCTAAGG | CTCGAGCAGCCTCACTGTTG |
| *DNAJB11* | NM_016306.5 | 141 | GTGATTGCCGGACGAGATTT | TTTCTCCTGGGCTTGTGGAT |
| *DNAJC10* | NM_018981.2 | 162 | TCGGCAAACACACTAGAGGA | TGGTGCAGAGGAACAGTCAA |
| *EDNRA* | NM_001957.3 | 154 | TGTAAAGGACTGGTGGCTCT | CACTTCTCGACGCTGCTTAA |
| *FBN2* | NM_001999.3 | 129 | GTCAGTCGCTCTTCATGCTG | GGGGTTAGGTCTGAAGCCTT |
| *FKBP14* | NM_017946.3 | 113 | ACGGCTCCTTATTTCACTCCAC | ACACATTCCTTTCAAGCCCT |
| **GAPDH*** | [NM_002046.4](http://www.ncbi.nlm.nih.gov/nuccore/NM_002046.4) | 109 | ATTCCACCCATGGCAAATTC | TTGATTTTGGAGGGATCTCG |
| **HPRT*** | [NM_001920.3](http://www.ncbi.nlm.nih.gov/nuccore/NM_001920.3) | 134 | TGACCAGTCAACAGGGGAC | GCCTGACCAAGGAAAGCAA |
| *HSPG2* | NM_001291860.1 | 122 | AGCAGAGTCCGACTGGCATC | GCAGGCTCCTGGAGAAGACA |
| *ITGA3* | NM_002204.3 | 142 | ATGGGTGAGTCTGGCATGAA | CATTGCTGACTTCGTAGGGC |
| *KIF11* | NM_004523 | 119 | CCCCGTAACAAGAGAGGAGT | AGAGTAGCTGCAGTTGTCCT |
| *KIF15* | NM_020242.2 | 117 | GACCAAACAGCAGGAAGAGC | AGGACTACTCGCAGGTCATG |
| *KIF18A* | NM_031217.3 | 157 | CAACTTGGACCAGTTCAGCC | GCTGCACACTTTGAGATGGT |
| *KIF20A* | NM_005733.2 | 140 | GCTCTGTCGTCTCTACCTCC | ACGGACACAACCCTGATCTT |
| *KIF2C* | NM_006845.3 | 63 | CTGCGAGATGTCATCAAGGC | TTGTCTGCTAGCCTGCTCTT |
| *KIF4A* | NM_012310.4 | 160 | TTTAATCCCGTCTGTGCCAC | TCCCTGGAGCCTTGTTTTCT |
| *LOXL3* | NM_032603.2 | 121 | GGAGCTCTCCCTCTGGAAGT | GAGTCGGATCCTGGTCTCTG |
| *MMP24* | NM_006690.3 | 83 | TGGACAGAAGTGGAGGCAAA | CTTTCCGCGTGTCTAGCTCA |
| *P4HA2* | NM_004199.2 | 160 | GTTTGGTGTCCTGAGCTGTG | CATTTTGTTGGCCCAGCTCT |
| *P4HA3* | NM_182904.4 | 71 | TGCAGAACCATGGCTACAGA | TGATGCGGTACTCCACTTGT |
| *PBK* | NM_018492.3 | 73 | TGGCTTTGGTACTGGGGTAA | AGCCCAAGGAGAATGAGACA |
| *PDIA4* | NM_004911.4 | 104 | GATGCCGCTAACAACCTGAG | TGCATTACAACCAACTGCCC |
| *PDIA5* | NM_006810.3 | 110 | CTTCTGGAAATGCGGCATCA | CACGCTGTACTCCTCCTTGA |
| *PDIA6* | NM_001282704.1 | 128 | TTTCTGATAACGCCCCACCT | TGCAGCTCCAGTATCAAGGA |
| *PLK1* | NM_005030.4 | 124 | GCCCCTCACAGTCCTCAATA | CTGCAGCATGTCACTGAGGT |
| *PSMA6* | NM_002791.2 | 172 | CTGCAGCGGGAGTTAAACAA | TCAACTGTCACTACTCCAACTTC |
| *PSMB6* | NM_002798.2 | 129 | CTGAATGAGCCTCCACTGGT | GTACACCTGCCCTCCTTCTT |
| *PSMC3* | NM_002804.4 | 110 | GCCCAACACCCAAGTTAAGG | TGGGCATCGGGAACTCTATC |
| *PSMD2* | NM_002808.4 | 129 | CCCGAATGCTGGTTACGTTT | TTGTATGCGTCTGGAACCCT |
| *TPX2* | NM_012112.4 | 121 | CCCAGAGAAAGCCAAGGGTA | CACCACCTCTTGCTGCATTT |
| *TXN* | NM_003329.3 | 106 | AGCAGATCGAGAGCAAGACT | ATCATTTTGCAAGGCCCACA |

*reference genes.
