# Supplementary material for: Transcriptome analysis of skin fibroblasts with dominant negative COL3A1 mutations provides molecular insights into the etiopathology of vascular Ehlers-Danlos syndrome
Source: PLoS One. 2018 Jan 18;13(1):e0191220. doi: 10.1371/journal.pone.0191220 (PMC5773204; doi:10.1371/journal.pone.0191220)
Supplement: S6 Table — (DOCX) [file pone.0191220.s006.docx]

| **S6 Table. Top canonical pathways perturbed in vEDS patients’ skin fibroblasts.** | | | |
| --- | --- | --- | --- |
| **Name** | **FDR 5%** | **Ratio^a^** | **DEGs** |
| **Cell cycle** | 7.81E-08 | 23/124 | ***CDC7, CDK1, SMAD3, TTK, PTTG1, MCM3, CDC26, CDC27, CDK2, MCM6, CCNB1, RAD21, MCM7, CDKN1B, CCNB2,*** *CDKN2B****, HDAC1, PLK1, ORC4, BUB1, ORC5, CCNA2, BUB3*** |
| **Systemic lupus erythematosus** | 1.33E-06 | 22/134 | ***HIST1H3J, HIST1H4L, HIST1H4K, SNRPD3, HIST1H2BH, SSB, HIST1H2BM, H2AFV, HIST1H3A, H2AFY2, HIST1H3B, H2AFZ, SNRPB, HIST1H4F, H3F3A, HIST1H4C, HIST1H4D, HIST1H3E, HIST1H3F, HLA-DOB, HIST1H3H, HIST1H3I*** |
| **DNA replication** | 2.42E-04 | 9/36 | ***PRIM1, RFC4, MCM7, POLE2, RFC1, SSBP1, RFC2, MCM3, MCM6*** |
| **Mismatch repair** | 0.004083 | 6/23 | ***MSH6, MSH2, RFC4, RFC1, RFC2, SSBP1*** |
| **Spliceosome** | 0.004536 | 15/133 | ***HNRNPA1L2, CHERP, SNRPD3, ALYREF, SNW1, CDC5L, PRPF18, HNRNPA1, PRPF4, EIF4A3, ISY1, SYF2, SNRPB, SLU7, PRPF38A*** |
| **Ribosome** | 0.005535 | 15/136 | ***RPL36A, RPL27A, MRPS5, MRPL30, MRPL21, MRPS18C, MRPL15, RPL23, RPS3A, RPL22, RPL9, RPL21, MRPL16, RPS12, RPS27A*** |
| **Proteasome** | 0.017741 | 7/44 | ***PSMC5, PSMB6, PSMA6, PSMB1, PSMC3, PSMA4, PSMD2*** |

^a^: input genes/genes in pathway; the down-regulated genes are reported in bold.
